# The Germ Freak's Guide to Outwitting Colds and Flu: Guerilla Tactics to Keep Yourself Healthy at Home, at Work, and in the World

**DOI:** 10.3201/eid1201.051309

**Published:** 2006-01

**Authors:** Kimberly Sessions

**Affiliations:** * Emory University, Atlanta, Georgia, USA

**Keywords:** Book Review, The Germ Freak's Guide to Outwitting Colds and Flu, Allison Janse, Charles Gerba

According to self-described "germ freak" Allison Janse, it's a dangerous world out there and I am not talking about Al Qaeda, anthrax in the mailbox, or Hurricane Katrina. The real danger, according to Janse, comes from elevator buttons (severe acute respiratory syndrome), escalator handrails (group B *Streptococcus*), subway platforms (*Aspergillus*), kitchen sinks (salmonella), loofah sponges (*Staphylococcus aureus*), and children's ball pits (*Escherichia coli*). She has a point, of course; everyday objects can transmit disease, but the value of her point is frequently lost in a hodgepodge approach that makes no distinction between serious but rare events, everyday avoidable ills, and the merely yucky.

In this book, Janse and her collaborator Charles Gerba use (sometimes badly misplaced) humor to alert us to the risks we run from everyday items like our kitchen cutting board ("If you have a choice between licking a cutting board or a toilet seat... pick the toilet seat" p. 50). The book does a nice job of addressing the overuse of antimicrobial drugs; encourages even blatant germ freaks to save their money and not buy antimicrobial soap for everyday use; is loaded with useful tips for reducing your family's vulnerability to sharing bugs of all sorts; provides a quick overview of the transmission, symptoms, and incubation period of some of the most common bugs (influenza virus, norovirus, cold viruses, and *E. coli*); and can induce a mania for handwashing among even the most hygiene-challenged. Unfortunately, the authors spend too much time on items that have no bearing on the transmission of colds and influenza, or anything else for that matter, and not nearly enough time providing detailed, "how-to" instructions.

Even less helpful is an entirely too flippant attitude toward the potentially valuable role that germ freaks can play in public health education. In a section entitled "Operation Germ Evasion," the authors provide a list of suggested responses that germ freaks should memorize, so they won't be caught off guard at a party when faced with ignorant comments from non–germ freaks. Two examples will suffice: 1) Non–germ freak comment, "I read about this hygiene hypothesis that says being too clean is causing increased illness." Suggested response, "I didn't think you knew how to read" (p. 29). 2) Non–germ freak comment, "Children who grow up in homes that are too clean are more likely to have asthma." Suggested response, "Then your kids are safe because your house is a real dump" (p. 29). These responses would not only give Miss Manners serious pause, they overlook a valuable opportunity to teach skills in addressing, and placing in proper context, the kernels of truth embedded in comments such as these. Rather than belittling non–germ freaks, the authors would have been better advised to give space to a balanced discussion.

Ultimately, the best audiences for this book are fellow germ freaks, who will enjoy the social validation it provides, and persons with enough preexisting savvy about infectious disease to sort out the helpful tips from the overly dramatic prose ("When you touch the shopping cart handle laden with *E. coli* and then sample the deli turkey, your life could literally be in your own hands" [p. 15]). In context, this book is entertaining and informative, but I would not recommend it for general consumption, without prior sanitization by the informed.

